# Diverse high-pressure chemistry in Y-NH_3_BH_3_ and Y–paraffin oil systems

**DOI:** 10.1126/sciadv.adl5416

**Published:** 2024-03-13

**Authors:** Alena Aslandukova, Andrey Aslandukov, Dominique Laniel, Yuqing Yin, Fariia Iasmin Akbar, Maxim Bykov, Timofey Fedotenko, Konstantin Glazyrin, Anna Pakhomova, Gaston Garbarino, Eleanor Lawrence Bright, Jonathan Wright, Michael Hanfland, Stella Chariton, Vitali Prakapenka, Natalia Dubrovinskaia, Leonid Dubrovinsky

**Affiliations:** ^1^ Bavarian Research Institute of Experimental Geochemistry and Geophysics (BGI), University of Bayreuth, Universitaetsstrasse 30, 95440 Bayreuth, Germany.; ^2^ Material Physics and Technology at Extreme Conditions, Laboratory of Crystallography, University of Bayreuth, 95440 Bayreuth, Germany.; ^3^ Centre for Science at Extreme Conditions and School of Physics and Astronomy, University of Edinburgh, Edinburgh EH9 3FD, UK.; ^4^ State Key Laboratory of Crystal Materials, Shandong University, Jinan 250100, China.; ^5^ Department of Physics, Chemistry and Biology (IFM), Linköping University, SE-581 83 Linköping, Sweden.; ^6^ Institute of Inorganic Chemistry, University of Cologne, Greinstrasse 6, 50939 Cologne, Germany.; ^7^ Deutsches Elektronen-Synchrotron DESY, Notkestr. 85, 22607 Hamburg, Germany.; ^8^ European Synchrotron Radiation Facility, BP 220, 38043 Grenoble Cedex, France.; ^9^ Center for Advanced Radiation Sources, University of Chicago, Chicago, IL 60637, USA.

## Abstract

The yttrium-hydrogen system has gained attention because of near-ambient temperature superconductivity reports in yttrium hydrides at high pressures. We conducted a study using synchrotron single-crystal x-ray diffraction (SCXRD) at 87 to 171 GPa, resulting in the discovery of known (two YH_3_ phases) and five previously unknown yttrium hydrides. These were synthesized in diamond anvil cells by laser heating yttrium with hydrogen-rich precursors—ammonia borane or paraffin oil. The arrangements of yttrium atoms in the crystal structures of new phases were determined on the basis of SCXRD, and the hydrogen content estimations based on empirical relations and ab initio calculations revealed the following compounds: Y_3_H_11_, Y_2_H_9_, Y_4_H_23_, Y_13_H_75_, and Y_4_H_25_. The study also uncovered a carbide (YC_2_) and two yttrium allotropes. Complex phase diversity, variable hydrogen content in yttrium hydrides, and their metallic nature, as revealed by ab initio calculations, underline the challenges in identifying superconducting phases and understanding electronic transitions in high-pressure synthesized materials.

## Teaser

Single-crystal XRD supported by theoretical calculations reveals the formation of seven yttrium hydrides at high pressures.

## INTRODUCTION

Near–room temperature superconductors can drastically affect many areas of technology (*1*–*3*). Because of recent advances in experimental techniques, namely, due to the improvements of the diamond anvil cells (DACs), it became possible to synthesize novel materials, including polyhydride phases, through a combination of high temperature and high pressure. Many hydrogen-rich materials [such as metal hydrides LaH_10_ (*4*), CaH_6_ (*5*), BaH_12_ (*6*), YH_6_ and YH_9_ (*7*, *8*), CeH_9_ (*9*), PrH_9_ (*10*), ThH_10_ (*11*), UH_7_ and UH_8_ (*12*)] and covalent polyhydrides [H_3_S (*3*)] were claimed to be high temperature (high-*T*_C_) superconductors under high pressure. However, not all of the claims are easy to justify in the absence of detailed information about the phase and chemical composition of products of chemical reactions taking place in the sample chamber of a DAC after laser heating (LH). Reliable conclusions concerning the superconductivity, i.e., values of measured *T*_C_, the isotopic effect, and the dependence of the superconducting transition with magnetic field, are problematic considering a variable hydrogen content in the same phase and/or the inhomogeneous products’ mixture (namely, the presence of phases other than the superconducting one), which can greatly affect the resistivity (*13*), magnetic susceptibility (*3*, *14*), and magnetic resonance measurements (*3*, *15*, *16*). Therefore, the results of the published *T*_C_ are still heavily disputed (*13*, *17*–*20*).

As promising high-*T*_C_ superconductors, high-pressure yttrium hydrides have been extensively studied. Under ambient conditions, two yttrium hydrides *cF*4-YH_2_ (*Fm*$\bar{3}$*m*) and *hP*2-YH_3_ (*P*6_3_/*mmc*) (*21*) are known (in this work, we use Pearson symbols, which refer to the arrangement of Y atoms only; hydrogen content inferred from the chemical formulas may refer to an experimentally determined, theoretically proposed, or empirically estimated amount of hydrogen atoms). At low pressures (10 to 25 GPa), *hP*2-YH_3_ undergoes a phase transition to *cF*4-YH_3_ (*22*), which also can be produced by cold hydrogenation of *cF*4-YH_2_ (*23*). The *cF*4-YH_3_ phase was found to be stable at ambient temperature under high pressures up to 325 GPa (*7*). At pressures above 200 GPa, the long treatment of YH_3_ with pressurized hydrogen without heating leads to the formation of tetragonal *tI*2-YH_4_ and cubic *cI*2*-*YH_6_ (*7*). Heating yttrium with hydrogen precursors at mild pressures (up to 50 GPa) results in the formation of *hP*3-YH*_x_* (*x* = 1.4 to 3) hydrides with variable H-content along with *cF*4-YH_3_ (*7*). At higher pressure, above 100 GPa, the high-temperature syntheses of yttrium hydrides with a higher hydrogen content [*tI*2-YH_4_ (*7*), *cI*2*-*YH_6_ (*7*), *oI*2-YH_7_ (*Imm*2) and *aP*2-YH_7_ (*P*1) (*8*), and *hP*2-YH_9_ (*P*6_3_/*mmc*) (*7*, *24*)] were reported and obtained from different precursors. Among them, based on experimental data, the *cI*2*-*YH_6_ and *hP*2-YH_9_ phases were determined to have high superconducting transition temperatures, *T*_C_ ∼ 224 K at 166 GPa (*8*) and *T*_C_ ∼ 243 K at 201 GPa (*7*), respectively. Moreover, up to date, several theoretical studies on the Y-H system were reported (*8*, *25*, *26*) and a variety of hydrides with a high hydrogen content, namely, *mC*2-YH_8_ (*25*), *cF*4-YH_9_ (*F*$\bar{4}$3*m*), *cF*4-YH_10_ (*Fm*$\bar{3}$*m*) (*8*), and *mC*4-YH_12_ (*C*2/*c*) (*25*), were predicted to be stable but not yet found in experiments. It is important to note that the chemical compositions and structures of only YH_2_ and YH_3_ have been unambiguously determined (*22*, *23*), whereas those of all other hydrides have been based on theoretical predictions and/or empirical estimations. It is also worth mentioning that the accuracy and limitations of these predictions may vary among different studies. Therefore, there are some inconsistencies between the predictions and experiments (i.e., many predicted Y-H compounds have not been found so far, predicted *T*_C_ notably different from reported ones, etc.), some puzzling results, such as negative resistance for YH_6_ at 183 GPa (*8*), and very different *T*_C_ onsets from experiment to experiment (*27*).

Complex chemical reactions, which occur upon the synthesis of hydrides at high pressures, often strongly complicate the analysis and interpretation of the results. In experiments conducted in DACs, carbon is unavoidable and upon LH can participate in the chemical reactions. In particular, it has been shown that in laser-heated DACs yttrium carbides appear along with yttrium hydrides (*28*). So far, structures of high-pressure yttrium hydrides mentioned above have been determined from powder x-ray diffraction (XRD) data with the assistance of computations based on various structure search algorithms. Powder XRD patterns of the reaction products obtained by LH yttrium in hydrogen-containing precursors (NH_3_BH_3_ or paraffin oil) (*7*, *8*, *11*, *29*, *30*) are often complex due to overlapping reflections from many compounds, which make phase identification and structural analysis difficult and ambiguous. Moreover, there are reports on still unknown phases whose structures could not be solved from powder XRD data (*7*, *8*).

Here, we present the results of the first single-crystal XRD (SCXRD) studies of the Y-NH_3_BH_3_ and Y–paraffin oil systems in the laser-heated DACs under high-pressure, high-temperature conditions up to ~170 GPa and ~3000 K. The extremely rich chemistry of the synthesized high-pressure yttrium compounds (hydrides and carbides) was observed. Besides two known solids (*cF*4-YH_3_ and *tI*2-YH_3_), five previously unknown yttrium hydrides (*hP*3-Y_3_H_11_, *hP*2-Y_2_H_9_, *cP*8-Y_4_H_23_, *hP*26-Y_13_H_75_, and *cF*80-Y_4_H_25_), two previously unknown yttrium allotropes (*hP*3-Y-II and *tI*8-Y), and an yttrium carbide (YC_2_) have been found. They were all characterized experimentally and through density functional theory (DFT) calculations. Our results demonstrate the broad compositional and structural variety of possible phases in Y-NH_3_BH_3_ and Y–paraffin oil systems.

## RESULTS

The details of the sample preparation, data collection, structure determination, and refinement are described in the Supplementary Materials (see Supplementary Text, fig. S1, and table S1). The hydrides were obtained in DACs via LH up to ~3000 K the starting material, i.e., yttrium, loaded along with one of two kinds of hydrogen-rich precursors—ammonia borane (NH_3_BH_3_) or paraffin oil (C_n_H_2n+2_)—which also act as pressure transmitting media. The use of these hydrogen sources has already been demonstrated to be an effective alternative to pure hydrogen for DAC synthesis experiments in many studies (*7*, *8*, *11*, *15*, *16*, *29*–*32*). Concerning the potential contamination of the system with carbon from paraffin oil, the use of pure H_2_ in experiments in laser-heated DACs does not bring a substantial advantage, as carbon from diamond anvils is always present in the system. Each sample was pressurized to the target pressure, laser-heated, and then characterized by in situ synchrotron SCXRD. The details of all DAC experiments and a list of obtained phases are provided in table S1. The positions of nonhydrogen atoms in the crystal structures of yttrium hydrides synthesized in this work were determined from synchrotron SCXRD data; the positions of H atoms could not be constrained from the SCXRD data, and therefore, hydrogen content was estimated from the volume per yttrium atom (see Methods). The possible models of hydrogen arrangement in yet unknown hydrides were analyzed with the help of the Endeavour software (*33*) and then refined by DFT calculations when possible (for more details, see Supplementary Text and fig. S2 in the Supplementary Materials).

### Yttrium allotropes

Structural transformations of yttrium upon compression to more than 180 GPa at room temperature are well described in the literature (*34*). A recent study of the high-pressure (up to ~50 GPa) behavior of Y and Y-H systems under high temperatures (*32*) showed that LH affects the experimental results and revealed the *hP*3-Y yttrium allotrope (hereafter, this phase will be referred to as *hP*3-Y-I) and its hydride. In this work, after LH of yttrium at pressures of 120 and 138 GPa (tables S2 and S3 and fig. S3), two novel yttrium allotropes (*hP*3-Y-II and *tI*8-Y) were found. The volume per yttrium atom for both novel allotropes perfectly agrees with previously published data on the yttrium’s equation of state (EoS) (fig. S4) (*34*). SCXRD data unambiguously exclude any other nonhydrogen atoms apart from Y in the structures of these two new phases, and the presence of a detectable amount of hydrogen in these materials is excluded based on density considerations.

The Y allotrope found at 120 GPa has a hexagonal symmetry (space group *P*$\bar{6}$*m*2). Its unit cell contains three Y atoms distributed over two Wyckoff positions (table S2). As the Pearson symbol of this phase is the same as of the previously known hexagonal yttrium allotrope (*hP*3-Y-I), we designate it as *hP*3-Y-II. The structure consists of tightly packed layers of Y atoms stacked in a “..AAB..” block-sequence manner along the *c* direction (fig. S3A). Distances between yttrium atoms vary from 2.679 to 2.788 Å at 120 GPa.

The yttrium allotrope found at 138 GPa has a tetragonal symmetry (space group *I*4/*mcm*). The Y atoms are located at the 8*h* Wyckoff site (Pearson symbol *tI*8*-*Y; table S3) and form channels along the *c* direction (fig. S3B; interatomic distances vary from ~2.49 to ~2.84 Å at 138 GPa). While the crystal structure of *tI*8-Y is quite unusual, especially for material synthesized at very high pressures, it is isostructural to the host sublattice of the incommensurate Bi-III phase (*35*). Moreover, such an arrangement of Y atoms was predicted for the host sublattice of a hypothetical host-guest structure of yttrium (*36*). The analysis of our SCXRD data does not reveal any detectable residual electron density in the channels.

The full structural relaxations with fixed (experimental) volume for both Y allotropes reveal a substantial difference between the calculated pressure value and the experimental one (table S2 and fig. S4). Nevertheless, when considering DFT relaxations above 70 GPa for all Y allotropes known in the literature at these pressures [*oF*16 and *hR*24 (*34*)], they exhibit the same inconsistency (fig. S4). This further supports our interpretation that these yttrium phases obtained in our experiments are pure.

As mentioned above, yttrium undergoes several phase transitions upon compression at ambient temperature (*34*). For the pressure range relevant to our study, DFT enthalpy calculations reproduce the well-known *hR*24-Y → *oF*16-Y phase transition at 106 GPa (fig. S5) (*34*). The previously unknown *hP*3-Y-II and *tI*8*-*Y yttrium allotropes are not thermodynamically stable with respect to the competing phases. Considering that at ~120 GPa the enthalpy difference of the previously unknown *hP*3-Y-II and *tI*8-Y yttrium allotropes with the known *oF*16-Y phase is significant (~0.20 and ~0.32 eV/atom, correspondingly; fig. S5) and cannot be explained only by the temperature contribution (*k*_B_*T* is equal to 0.259 eV at 3000 K), the reason for the formation of the two phases, *hP*3-Y-II and *tI*8-Y, remains unclear.

**Fig. 1.**
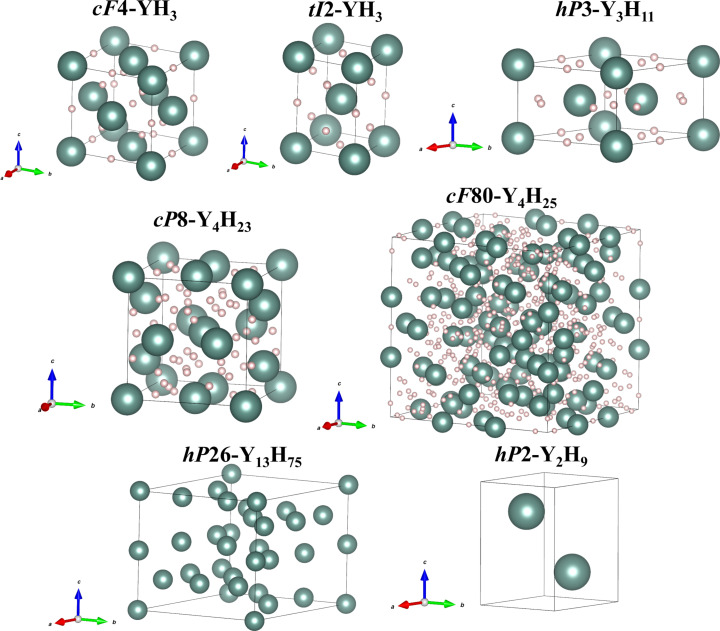
The crystal structures of the yttrium hydrides synthesized in this work. Yttrium and hydrogen atoms are shown in green and light pink, respectively. For Y_2_H_9_ and Y_13_H_75_, only the Y metal framework is shown.

The improvement of modern synchrotron x-ray sources and the recently developed methodology of diffraction analysis of polycrystalline samples allowed us to find two known and five previously unknown yttrium hydrides apart of the yttrium allotropes described above (table S1 and Fig. 1). They could form due to temperature gradients during LH and due to possible differences in hydrogen diffusion into the depth of the metal in different samples. A detailed description of these compounds is given below.

### Previously known yttrium hydrides: YH_3_ and YH_4_

The previously known cubic yttrium hydride *cF*4-YH_3_ (Fig. 1 and table S4) was obtained after sample LH, regardless of the hydrogen precursor, at five different pressures: 87, 90, 100, 116, and 120 GPa. The crystallographic data and refinement parameters at all pressures are summarized in table S4. Phonon dispersion calculations in the harmonic approximation showed the dynamical stability of this phase in the whole studied pressure range (fig. S6A). The pressure dependence of the unit cell volume is shown in fig. S7 and is in a good agreement with the DFT calculations. The mutual agreement of experimental and theoretical results allows us to conclude that the structural model of *cF*4-YH_3_ (i.e., the location of hydrogen atoms) and the used Vienna Ab initio Simulation Package (VASP) potentials are correct (see Methods for computational details).

Tetragonal yttrium hydride (*I*4/*mmm*, *tI*2-YH_3_; Fig. 1) was found at five different pressures: 87, 90, 100, 116, and 120 GPa (table S5). Yttrium atoms occupy the 2*a* Wyckoff position (0; 0; 0) in the nodes of the body-centered tetragonal lattice. Two phases with different stoichiometries, *tI*2-YH_3_ (*8*, *37*) and *tI*2-YH_4_ (*7*, *8*), were reported to have similar Y atom arrangement in structures with different *a*/*c* ratios: 0.6819 and 0.5303 at 150 GPa, respectively. The experimentally determined pressure dependence of the unit cell volume of the hydrides is shown in fig. S7. For experiments in which paraffin was used as a hydrogen source, there is a good agreement with the DFT results for the *tI*2-YH_3_ model, whereas, for the experiment with ammonia borane, the DFT calculated unit cell volume is much closer to that corresponding to the YH_4_ composition. However, the *a*/*c* ratios for all experimental *tI*2 phases are similar within the experimental uncertainties and differ a lot from values reported for the YH_4_ phase (*7*, *8*). Thus, the differences in the volume per yttrium atom among *tI*2 yttrium hydrides (fig. S7) could be explained by the variable hydrogen content depending on hydrogen precursors. The cubic phase synthesized in the DACs with paraffin oil has a volume per Y atom systematically smaller than the tetragonal one (fig. S7) that suggests a lower hydrogen content, which can be reflected in the chemical formula as YH_3-δ_. The *tI*2 phase obtained from ammonia borane has a larger atomic volume and is thus expected to have the YH_3+δ_ composition. For an ideal YH_3_ stoichiometry, theoretical calculations show no difference in volume and enthalpy between the *cF*4 and *tI*2 phases. Phonon calculations demonstrate that hydrides with the YH_3_ composition are dynamically stable at all experimental pressures (fig. S6, A and B), while YH_4_ can be stabilized only at 140 GPa (fig. S6, C and D), which is in good agreement with previously published experimental results, in which this phase was seen above 135 GPa (*7*, *8*).

### Previously unknown yttrium hydrides synthesized in this work

The analysis of SCXRD data revealed five phases with a so far unknown arrangement of yttrium atoms. The shortest Y-Y distances in each of the five previously unknown yttrium-based compounds are remarkably larger than those in pure yttrium metal under the same conditions (table S6). Neither boron, carbon, nor nitrogen was detected by SCXRD data analysis in these structures, which allows classifying the novel phases as hydrides.

For these yttrium hydrides, the hydrogen contents were estimated using the “Retger’s law” approximation, which is based on the empirical linear relationship between composition and the unit cell volume (fig. S8; for more details, see the Supplementary Materials). The obtained hydrides’ stoichiometries were then used for proposing structural models. For this purpose, we applied our original approach involving the application of Endeavour software and DFT calculations: Possible arrangements of hydrogen atoms for given stoichiometries were proposed by Endeavour, and the following validation of the structure models was done by DFT. The details of this approach are described explicitly in Supplementary Text.

The reaction between Y and paraffin oil at 87 GPa and between Y and ammonia borane at both 90 and 120 GPa led to the formation of the *hP*3-Y-I (*32*)–based hydride (space group *P*6*/mmm*; Fig. 1 and table S7). At 120 GPa, it has a lattice parameter of *a* = 4.978(3) Å and *c* = 3.0784(15) Å [*V* = 66.06(9) Å^3^]. The *hP*3-YH*_x_* hydrides with variable stoichiometry (*x* = 2.4..3) have been reported, but the arrangement of hydrogen atoms has not been proposed yet (*32*). In this study, the estimated stoichiometry at all experimental pressures is Y_3_H_11_ = YH_3.77_ (fig. S8, B and C), which differs from previously published results at lower pressures (*32*). A possible structural model for the Y_3_H_11_ hydride is shown in Fig. 1 and fig. S9, and the corresponding crystallographic data are summarized in table S8.

There are a few yttrium hydrides, which we observed only at a single pressure point (table S9). The YH_4.5_ hydride, whose stoichiometry in integer numbers is Y_2_H_9_, was obtained at 120 GPa (Fig. 1 and table S9). Yttrium atoms have the hexagonal close-packed (*hcp*) arrangement (*P*6_3_/*mmc*, *hP*2). The lattice parameters at 120 GPa are *a* = 3.162(3) Å and *c* = 4.958(2) Å [*V* = 42.94(9) Å^3^]. The same *hcp* yttrium framework was reported in *hP*2-YH_9_ synthesized above 180 GPa (*7*); however, according to Retger’s law approximation, in our experiment, the volume per atom suggested half as less hydrogen content. It is impossible to find a reliable DFT-verified crystal structure of Y_2_H_9_, because the lattice symmetry does not allow placing an odd number of hydrogen atoms with full chemical occupancy in the unit cell, so one can assume either partial occupancy of some hydrogen positions or a symmetry reduction due to the hydrogen sublattice.

At 138 GPa, after LH yttrium in paraffin oil, two previously unknown yttrium hydrides were found: YH_5.77_ (Y_13_H_75_, the closest stoichiometry in integer numbers) and YH_5.75_ (Y_4_H_23_) (Fig. 1 and table S9). The structure formed by the Y atoms in Y_13_H_75_ has a hexagonal symmetry (space group *P*6_3_/*mmc*, *hP*26). At 138 GPa, it has the lattice parameters of *a* = 8.9730(13) Å and *c* = 8.9085(8) Å [*V* = 621.17(19) Å^3^]. The DFT structure relaxation of several of the created structural models of the hydride with the Y_13_H_75_ composition resulted in a high disagreement with experimental data and showed dynamical instability of all of them. Thus, there is no reliable model for the H atoms’ location in the structure of Y_13_H_75_ yet.

**Fig. 2.**
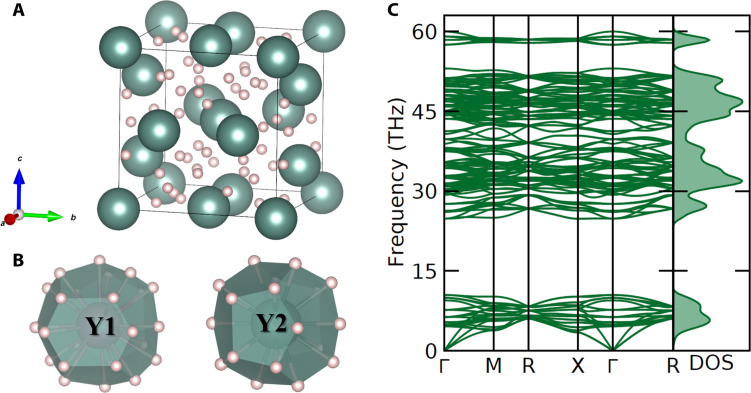
Crystal structure and the results of DFT phonon calculations for the *cP*8-Y_4_H_23_ yttrium hydride. (**A**) Compound’s unit cell, with the Y atoms shown as green and hydrogen atoms as light pink balls. (**B**) Coordination environment of the Y1 and Y2 atoms in Y_4_H_23_. (**C**) Phonon dispersion curves along high-symmetry directions in the Brillouin zone and phonon density of states for Y_4_H_23_ calculated at 138 GPa.

According to our calculations, the Y_4_H_23_ hydride adopts the Na_4_Si_23_-type structure (*Pm*$\bar{3}$*n, cP*8) (Fig. 1 and table S9), which has previously been experimentally seen and/or theoretically predicted for the three metal hydrides *M*_4_H_23_, where *M* = Ba (*38*), La (*31*), and Eu (*39*, *40*). The DFT structure relaxation shows a very good agreement with the experimental data (table S10), and *cP*8-Y_4_H_23_ was found to be dynamically stable at the experimentally produced pressure of 138 GPa (Fig. 2C). This compound has two isolated H atoms (H1 and H3) and hydrogen dimers H2-H2 with an intramolecular bond length of 0.95 Å; therefore, one can suggest that the electronic state of these two kinds of hydrogen atoms has to be very different. For a deeper understanding of the electronic properties of Y_4_H_23_, we calculated the electronic band structure and the electronic density of states (eDOS) at the synthesis pressure of 138 GPa. It was found that this compound exhibits metallic properties and the main contribution at the Fermi level comes from the Y *d*-states, while hydrogen makes quite small contributions (fig. S10A). Previously, it was shown that strong Y-H hybridization could be responsible for the superconductivity in *cF*4-YH_3_ and *cI*2-YH_6_ (*26*). For Y_4_H_23_, there is a negligible overlap of Y- and H-eDOS (no strong Y-H hybridization), and therefore, this compound is not expected to be a high-temperature superconductor (*26*).

The Y_4_H_25_ (= YH_6.25_) compound was synthesized upon laser-heating yttrium and paraffin oil at 171 GPa (Fig. 1 and table S11). Yttrium atoms form a cubic structure (space group *F*$\bar{4}$3*m*, *cF*80) with a large unit cell [*a* = 12.4201(14) Å, *V* = 1915.9(4) Å^3^]. The full relaxation of the obtained hydride shows perfect agreement with the experimental data (table S11). Hydrogen triatomic units found in this compound have an intramolecular bond length of 0.93 Å, which is shorter than the H-H distance of the dimers constituting Y_4_H_23_. The calculations of eDOS at the synthesis pressure of 171 GPa showed the metallic nature of *cF*80-Y_4_H_25_, with the ratio of hydrogen/yttrium’s partial density of states at the Fermi level being higher than that in *cP*8-Y_4_H_23_ (fig. S10B).

The yttrium hydride *cI*2-YH_6_ (*Im*$\bar{3}$*m*), widely discussed in the literature (*7*, *8*, *24*–*26*), was not observed in this work. As reported in (*7*), YH_6_ was obtained by keeping YH_3_ in a hydrogen pressure medium at room temperature and pressures of over 100 GPa for a dozen hours. Calculated convex hulls of the Y-H system (*8*) showed that *cI*2-YH_6_ is metastable even at 150 GPa and lays 30 meV per atom above the convex hull. This may mean that yttrium hexahydride was observed in the experiments below 150 GPa (*7*, *24*) only due to the temperature factor. Our calculations of the convex hull for phases with available structural models at 90, 120, 138, and 171 GPa showed that Y_3_H_11_, Y_4_H_23_, and Y_4_H_25_ lay slightly above the convex hull (fig. S11). Among them, Y_4_H_23_ is preferable at all pressures. At 150 GPa, the hydrides with a higher hydrogen content, *aP*2-YH_7_, *cF*4-YH_9_, and *hP*2-YH_9_, were predicted to be stable (*8*). The hydride synthesized in this work with the highest hydrogen content, *cF*80-Y_4_H_25_, was the only phase found at 171 GPa, and the calculated convex hull suggests that it is thermodynamically not preferable. The temperature contribution to the energy, *k*_B_*T* equal to 0.259 eV at 3000 K, could play an important role in the hydride’s formation, and rapid quenching down to room temperature could stabilize the metastable phase.

**Fig. 3.**
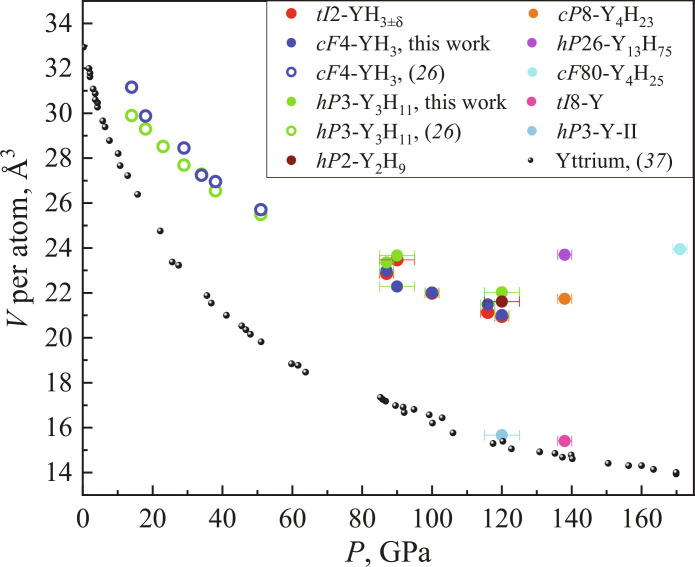
The pressure dependence of the volume per Y atom in the range of 1 bar to 170 GPa for the Y allotropes and the hydrides as determined from the experimental data. The data for the hexagonal *hP*3-Y-II and tetragonal *tI*8-Y allotropes, for *tI*2-YH_3±δ_, *cF*4-YH_3_, *hP*3-Y_3_H_11_, *hP*2-Y_2_H_9_, *cP*8-Y_4_H_23_, *cF*80-Y_4_H_25_, and *hP*26-Y_13_H_75_, which were obtained in this work, are shown in red, blue, green, brown, orange, cyan, and violet circles, respectively. The experimental pressure dependence of the volume per Y atom for yttrium allotropes published in (*34*) is shown in small black circles; the experimental values for *cF*4-YH_3_ and *hP*3-Y_3_H_11_ from (*32*) are shown as open blue and green symbols, respectively. Pearson symbols refer to the arrangement and content of Y atoms only.

The idea that with increasing pressure the H:Y ratio should increase is common in the literature (*8*, *31*). Our results support this idea: The hydrogen content per yttrium atom increases from 3 at 87 GPa (YH_3_, both *cF*4 and *tI*2) to 6.25 at 171 GPa (*cF*80-Y_4_H_25_) (Fig. 3).

### Yttrium carbide

Apart from hydrogen, paraffin oil and ammonia borane also contain carbon, nitrogen, and boron, which can react with yttrium, resulting in the formation of various yttrium compounds. In our experiments at 87 and 120 GPa with paraffin oil, we found a previously unknown yttrium carbide, YC_2_.

In the structure of YC_2_ (*P*6_3_/*mmc*, *hP*2), the yttrium atoms occupy the 2*c* Wyckoff position (1/3; 2/3; 3/4) forming a hexagonal close packing. The carbon atoms C1 and C2 are located at the 2*a* (0; 0; 0) and 2*d* (1/3; 2/3; 1/4) Wykoff positions, respectively (fig. S12A). The lattice parameters at 120 GPa are *a* = 3.600(17) Å and *c* = 4.439(8) Å [*V* = 49.82(13) Å^3^]. A similar metal-carbon framework was reported for LaCH_2_ synthesized at 96 GPa (*31*); however, our detailed analysis showed that the high remaining electronic density at the 2*d* position can be better described as an additional carbon atom C2, instead of H_2_ units. Y is surrounded by 11 carbon atoms (6·C1 and 5·C2), with an average Y─C bond length of 2.25 Å (fig. S12B). The unit cell parameters, atomic coordinates, and pressure obtained from DFT structure relaxation agree with the experimental data (table S12).

## DISCUSSION

To summarize, in this work, the complexity of the chemical processes in the Y-NH_3_BH_3_ and the Y–paraffin oil systems at high pressures and high temperatures was proven due to the analysis of products of chemical reactions in DACs after LH, which revealed inhomogeneous mixtures of various phases at a pressure range of 87 to 171 GPa. Seven yttrium hydrides YH*_x_* (*x* = 3 to 6.25), two previously unknown yttrium allotropes, and one carbide YC_2_ were detected and characterized using synchrotron SCXRD. The hydrogen content in the discovered hydrides was found to increase with pressure, i.e., from YH_3_ at 87 GPa to YH_6.25_ at 171 GPa.

Theory is not yet able to predict all possible hydride and nonhydride phases, and powder XRD is prone to missing some reaction products in laser-heated DACs. The presence of phases other than yttrium hydrides, especially compounds with metallic conductivity, can lead to a sharp drop in the electrical resistance of a sample upon low-temperature measurements and the misinterpretation of the results. Therefore, knowledge of the phase composition, crystal structures, and transport properties of individual phases is needed for unambiguous judgment on the electronic properties of complex hydride systems. Our results point out substantial difficulties in producing monophase samples using paraffin oil and NH_3_BH_3_ precursors, which are necessary for a reliable assessment of the physical properties of materials, including superconductivity, and promote the use of SCXRD on polycrystalline samples as an essential tool for hydrides’ characterization.

## METHODS

### Sample preparation

The BX90-type large x-ray aperture DAC equipped with Boehler-Almax–type diamonds (culet diameter of DAC1, DAC2, DAC3, and DAC5 is 120 μm, while that of DAC4 is 80 μm) was used for SCXRD studies (*41*, *42*). Rhenium foil preindented to a thickness of ~20 or ~13 μm and a hole of ~60 or ~40 μm in diameter drilled in the center of the indentation served as a sample chamber, for 120- and 80-μm culets, respectively. A piece of yttrium was placed in the sample chamber filled with paraffin oil (DAC1 to DAC4) or NH_3_BH_3_ (DAC5). Pressure was determined using the EoS of Re (*43*, *44*) and additionally monitored by the Raman signal from the diamond anvils (*45*). Samples were compressed up to their target pressure and laser-heated to ~3000(200) K. LH of the samples was performed using an in-house LH setup (*46*), equipped with two yttrium-aluminum-garnet (YAG) lasers (1064 nm central wavelength) and an IsoPlane SCT 320 spectrometer with a 1024 × 2560 PI-MAX 4 camera for collection of thermal emission spectra from the heated spot. Temperatures were estimated by fitting of thermal emission spectra of the sample to the gray body approximation of Planck’s radiation function over a given λ range (570 to 830 nm).

### XRD measurements

XRD measurements for DAC1 (at 87 GPa) and DAC4 (at 138 GPa) were performed at the ID11 beamline of the European Synchrotron Radiation Facility (ESRF; Grenoble, France) with the x-ray beam (λ = 0.2844 Å) focused down to 0.75 μm × 0.75 μm, and data were collected with the Eiger2X CdTe 4M hybrid photon-counting pixel detector. XRD measurements for DAC5 (at 90 GPa) and DAC4 (at 171 GPa) were performed at the ID27 beamline of the ESRF with the x-ray beam (λ = 0.3738 Å) focused down to 1.5 μm × 1.5 μm. DAC5 (at 120 GPa) was measured at the ID15B beamline (ESRF) with an x-ray beam (λ = 0.4103 Å) focused to a size of 4 μm × 4 μm. At ID27 and ID15B, the XRD patterns were collected on an Eiger2X CdTe 9M hybrid photon-counting pixel detector. XRD measurements for DAC2 and DAC3 were done at the GSECARS 13IDD beamline of the Advanced Photon Source (APS; Lemont, USA; λ = 0.2952 Å, 2 μm × 2 μm), and data were collected with Pilatus 1M detector. The data for DAC1 (at 116 GPa) were collected at the P02.2 beamline of Petra III [Deutsches Elektronen-Synchrotron (DESY), Hamburg, Germany] with the x-ray beam (λ = 0.2882 Å) focused down to 1.8 μm × 2 μm by a Kirkpatrick-Baez mirror system, and diffraction patterns were collected on a PerkinElmer 1621 XRD flat-panel detector.

For SCXRD measurements, samples were rotated around the vertical ω axis in a range of ±36°. The XRD images were collected with an angular step Δω = 0.5°. The CrysAlisPro software package was used for the analysis of the SCXRD data (indexing, data integration, frame scaling, and absorption correction) (*47*). A single crystal of (Mg_1.93_,Fe_0.06_)(Si_1.93_,Al_0.06_)O_6_ orthoenstatite [*Pbca*, *a* = 18.2391(3), *b* = 8.8117(2), *c* = 5.18320(10) Å] was used to calibrate the instrument model of the CrysAlisPro software (sample-to-detector distance, the detector’s origin, offsets of the goniometer angles, and rotation of the x-ray beam and the detector around the instrument axis). The DAFi program (*48*) was used for the search of reflections’ groups belonging to individual single-crystal domains. Using the OLEX2 software package (*49*), the structures were solved with the ShelXT structure solution program (*50*) using intrinsic phasing and refined with the ShelXL (*51*) refinement package using least-squares minimization. The procedure of the analysis of the SCXRD data is described explicitly in Supplementary Text in the Supplementary Materials. Crystal structure visualizations were made with the VESTA software (*52*). The experimental EoSs were obtained by fitting the pressure-volume data using the EoSFit7-GUI (*53*).

### Computational details

The first-principles calculations were done using the framework of DFT as implemented in the VASP (*54*). To expand the electronic wave function in plane waves, we used the projector-augmented-wave (PAW) method (*55*, *56*). The generalized gradient approximation (GGA) functional was used for calculating the exchange-correlation energies, as proposed by Perdew-Burke-Ernzerhof (PBE) (*57*). The PAW potentials with following valence electrons of 4*s*4*p*5*s*4*d* for Y, 1*s* for H (the potential “H_h”-harder than the standard potential), and 2*s*2*p* for C were used. The Monkhorst-Pack (*58*) *k*-point grid and an energy cutoff for the plane wave expansion were selected on the basis of convergence tests with a threshold of 2 meV per atom for energy and 1 meV/Å per atom for forces. The phonon frequencies and phonon band structure calculations were performed in the harmonic approximation with the help of the PHONOPY software (*59*) using the density-functional-perturbation theory (DFPT) for yttrium hydrides. The tetrahedron method was used for Brillouin zone integrations (*60*). EoS and static enthalpy calculations were performed via variable-cell structural relaxations between 70 and 170 GPa. In our calculations, temperature, configurational entropy, and the entropy contribution due to lattice vibrations were neglected.
