# Supplementary material for: Diverse high-pressure chemistry in Y-NH3BH3 and Y–paraffin oil systems
Source: Sci Adv. 2024 Mar 13;10(11):eadl5416. doi: 10.1126/sciadv.adl5416 (PMC10936948; doi:10.1126/sciadv.adl5416)
Supplement: Supplementary file 1 — Supplementary Text Figs. S1 to S12 Tables S1 to S12 Legends for data S1 to S8 [file sciadv.adl5416_sm.pdf]

Science Advances
AAAS

Supplementary Materials for

Diverse high-pressure chemistry in Y-NH_3BH_3 and Y–paraffin oil systems

Alena Aslandukova *et al.*

Corresponding author: Alena Aslandukova, alena.aslandukova@uni-bayreuth.de

Sci. Adv. **10**, eadl5416 (2024)
DOI: 10.1126/sciadv.adl5416

The PDF file includes:

Supplementary Text
Figs. S1 to S12
Tables S1 to S12
Legends for data S1 to S8

Other Supplementary Material for this manuscript includes the following:

Data S1 to S8

Supplementary Text

<u>Discussion 1. Details of the data acquisition and processing for synchrotron single-crystal X-ray diffraction in DACs from laser-heated polycrystalline samples</u>

The high-pressure high-temperature reactions in DACs usually result in multigrain and often multiphase samples. On the synchrotron facilities providing sufficiently small X-ray beam we can study such polycrystalline samples *in situ*, applying methods of SCXRD to individual grains of micrometer to sub-micrometer size. The procedure is briefly described below.

First, we conduct an XRD mapping of the sample chamber to locate various phases within the laser-heated spot. The phases are assigned based on single-crystal XRD and are shown in the resulting 2D XRD map in different colors. In this procedure, XRD is collected stepwise with a step (Δ) equal to the beam size. The number of steps should be sufficient to cover the entire sample chamber.

Figure S1a shows an example of a 2D XRD map for a sample in the chamber of ~30 μm in diameter with a diamond culet size of 80 μm. The map is built upon 961 frames (31x31 points separated by Δ = 1 μm in each direction). The map enables also to pinpoint the location of grains (domains) of the best crystallinity for a particular phase, those giving the richest spotty pattern, thus most appropriate for SCXRD measurements.

The SCXRD is then measured in the selected points while the DAC is rotated around the vertical goniometer axis ω with the frames being collected from ω = -36° to ω = 36° with a step of Δω = 0.5°. A single frame from the SCXRD dataset at ω = 0° is shown in Fig. S1b. After the collection of the dataset for the selected point, it is analyzed in the CrysAlis^{Pro} software to identify phases and their crystal structures and chemical composition. The general procedure of the multigrain dataset analysis contains the following steps:

- Step 1. Perform the peak search using CrysAlis^{Pro} package. Figure S1c shows the reciprocal space representing all reflections of the dataset for the example under consideration. These can be the reflections from reaction products, initial reagents, pressure-transmitting medium, diamonds, and gasket material.
- Step 2. Apply ‘advanced filtering’ to get rid of diamond peaks and artifacts.
- Step 3. Find all single-crystal domains in the dataset using DAFi software (Fig. S1d).
- Step 4. Find the lattice parameters of all domains in the dataset using the indexing procedure implemented in CrysAlis^{Pro}.

- Step 5. Select the most strongly diffracting domain for each found phase (Fig. S1e), perform integration, and extract the *.hkl* file.
- Step 6. Solve and refine the crystal structure of each of the different phases found using Olex2 software.

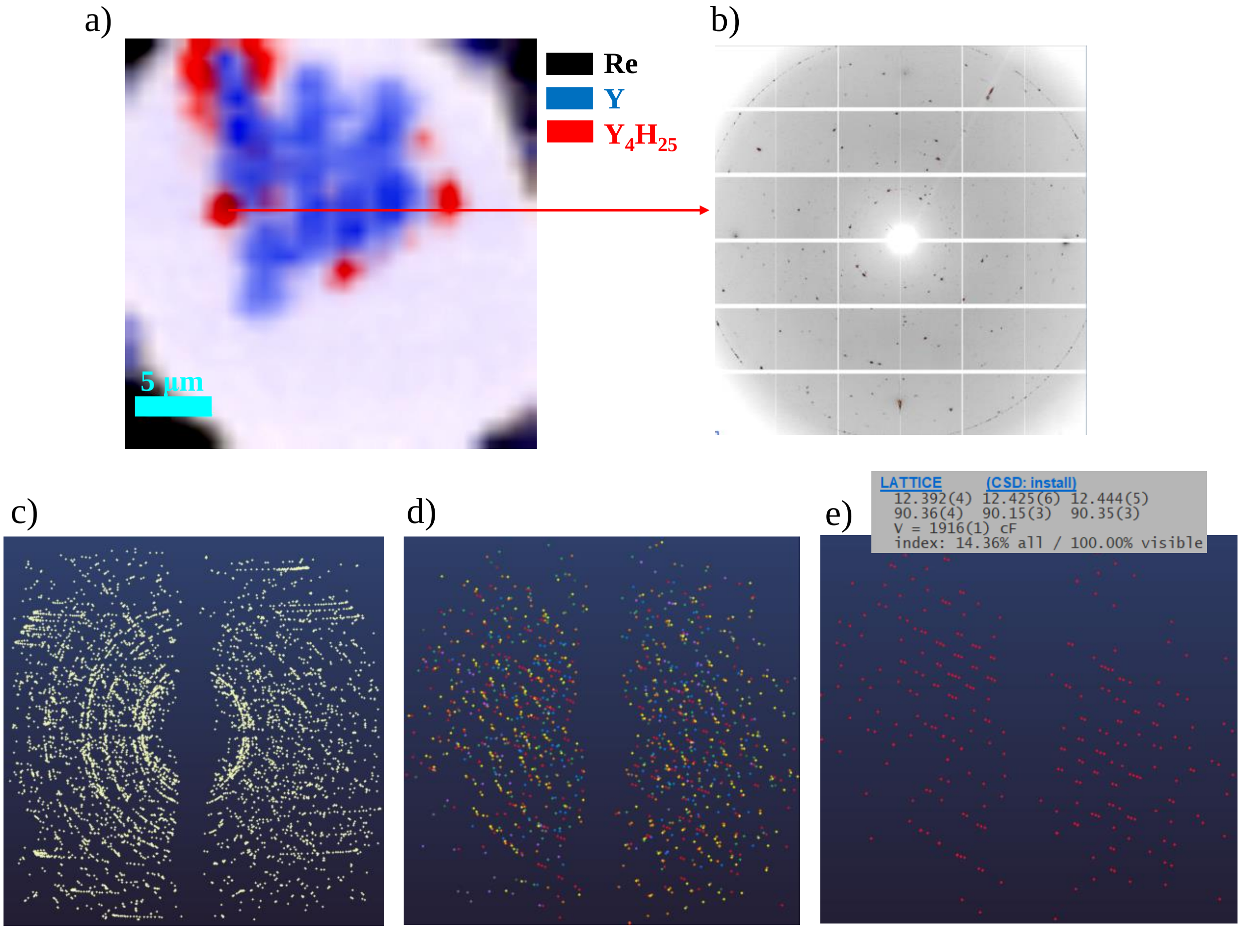

Fig. S1. Illustration of the procedure of the SCXRD data collection from a polycrystalline sample and the data analysis on example of *cF*80-Y_4H_{25} found in a DAC at 171 GPa. a) A 2D XRD map (31x31, Δ=1 µm) of the sample chamber showing the distribution of three phases (coloured in black, red, and blue) in the DAC. Phases assignment is based on single-crystal XRD. b) One frame from the SCXRD dataset taken at $\omega = 0°$. c) All collected reflections of a full dataset displayed in the reciprocal space. d) Eighteen sets of reflections from different domains (marked with different colours) found in this dataset using DAFi software. e) Reflections of the strongest *cF*80-Y_4H_{25} domain found by the DAFi program, for which integration and structure solution were done.

Structural description of novel yttrium allotropes

The hexagonal *hP*3-Y-II ($P\bar{6}m2$) was obtained at 120 GPa after laser heating Y in paraffin oil. At 120 GPa, the unit cell parameters are a = 2.773(4) Å and c = 7.06(2) Å (V = 47.0(2) Å^3). Yttrium atoms occupy two Wyckoff positions: Y1 at 1*a* (0; 0; 0) and Y2 at 2*i* (2/3; 1/3; 0.3043) (Table S2). Y-Y distances vary from 2.679 Å to 2.788 Å.

Tetragonal *tI*2-Y (*I*4/*mcm*) was found at 138 GPa, the unit cell parameters a = 5.3001(10) Å and c = 4.391(3) Å (V = 123.35(8) Å^3) (Table S3). Y atoms are located at 8*h* Wyckoff site (0.17029; 0.67029;0.5).

Structural description of yttrium hydrides

In the cubic *cF*4-YH_3 (space group $Fm\bar{3}m$, Pearson symbol *cF*4 refers to the structure formed by Y atoms), the yttrium atoms occupy the 4*a* Wyckoff position (0; 0; 0) forming the *fcc* lattice. Hydrogen atoms were placed to all octahedral (4*b* Wyckoff position (0.5; 0.5; 0.5)) and tetrahedral voids (8*c* Wyckoff position (0.25; 0.25; 0.25)) according to literature data (*23*).

In tetragonal yttrium hydride (*I*4/*mmm*, Pearson symbol *tI*2 refers to the structure formed by Y atoms) yttrium atoms occupy the 2*a* Wyckoff position (0; 0; 0). This tetragonal structure was reported to have two stoichiometries YH_3 (*8, 37*) and YH_4 (*7, 8*), with different a/c ratio. In the YH_3 hydrogen atoms occupy 4*d* (0; 0.5; 0.25) and 2*b* (0; 0; 0.5) Wyckoff positions, while in the YH_4 hydrogens are located at 4*e* (0; 0; 0.371) and 4*d* (0; 0.5; 0.25) Wyckoff positions.

The structural models of five novel yttrium hydrides were obtained using an original procedure involving the application of Endeavour™ software and DFT calculations (for more details see discussion 2). They may be not unique, but these models allow us to describe experimental and theoretical results in the best way.

The *hP*3-Y_3H_{11} hydride (space group of Y lattice is *P*6*/mmm*, *hP*3) was observed at 87, 90, and 120 GPa. Yttrium atoms occupy two Wyckoff positions: 1*a* (0; 0; 0) and 2*d* (1/3; 2/3; 1/2). In our proposed structural model, the hydrogen atoms are located at the 2*c* (0.5; 0; 0), 3*f* (2/3; 1/3; 0), and 6*k* (0.24; 0; 0.5) Wyckoff positions. The full structural relaxation perfectly reproduces experimental observations (Table S8). In the structure of *hP*3-Y_3H_{11} yttrium atoms Y1 are surrounded by 8 hydrogen atoms, while Y2 atoms have 18 hydrogen atoms in their first coordination sphere (Fig. S9). This compound has only isolated H atoms because the shortest H-H distance of ~1.13 Å at 120 GPa, that is too long for hydrogen H_2 molecule.

The *hP*2-Y_2H_9 was observed at 120 GPa. Yttrium atoms occupy the 2*c* Wyckoff position (1/3; 2/3; 1/4) forming the *hcp* lattice ($P6_3/mmc$, *hP*2). The lattice parameters at 120 GPa are a = 3.162(3) Å and c = 4.958(2) Å (V = 42.94(9) Å^3). The structural model of hydride with the Y_2H_9 composition was not suggested.

The lattice formed by yttrium in the structure of *hP*26-$Y_{13}H_{75}$ has hexagonal symmetry (space group $P6_3/mmc$, *hP*26). At 138 GPa, it has the unit cell parameters of a = 8.9730(13) Å and c = 8.9085(7) Å (V = 621.17(19) Å^3). Yttrium atoms occupy four crystallographically independent positions: 2*a* (0; 0; 0), 12*k* (0.2041; 0.4083; 0.5629), 6*h* (0.1186; 0.2373; 0.25), and 6*h* (0.4558; 0.9116; 0.25). The analysis of the data from the ICSD showed that this arrangement of metal atoms is known in Hf_9Mo_4B structure type (ICSD 23788) – Y occupies the positions of both Hf and Mo. The structural model of hydride with the $Y_{13}H_{75}$ composition was not suggested.

The *cP*8-Y_4H_{23}, which was obtained at 138 GPa, has a cubic structure ($Pm\bar{3}n$, *cP*8) made of two types of Y atoms: Y1 at 6*d* (0.25; 0.5; 0) and Y2 at 2*a* (0; 0; 0) Wyckoff positions. Y2 atoms form a *bcc* lattice and have twelve Y1 neighbors, each located at the same distance of 3.12043(12) Å at 138 GPa (Table S10). The shortest metal-metal distance is $d_{Y1\text{-}Y1}$ = 2.791 Å. Hydrogen atoms in a suggested structure occupy three different Wyckoff positions: 6*c* (0.25; 0; 0.5), 16*i* (0.201; 0.201; 0.201), and 24*k* (0; 0.3103; 0.1492). The Y2 – H polyhedron is almost a perfect dodecahedron consisting of twelve H_5 pentagons with two types of Y-H distances: $d_{Y2\text{-}H2}$ = 1.921 Å –and $d_{Y2\text{-}H3}$ = 1.943 Å (Fig. 2b). The Y1 atoms surrounded by 24 hydrogen atoms, with Y-H distances ranging between 1.939 and 2.029 Å (Fig. 2b). This compound has isolated H atoms (H1 and H3) as well as hydrogen dimers H2-H2 with an intramolecular bond length of 0.95 Å.

The *cF*80-Y_4H_{25} was found at 171 GPa. Yttrium atoms form a cubic lattice ($F\bar{4}3m$, *cF*80) with a big unit cell (a = 12.4184(11) Å, V = 1915.1(5) Å^3) (Table S11). Y located at four Wyckoff positions: 24*f* (0.3130; 0; 0), 16*e* (0.3368; 0.3368; 0.3368), 24*g* (0.5616; 0.25; 0.25), and 16*e* (0.0943; 0.0943; 0.0943). The framework of yttrium atoms resembles the inverted sublattices of Dy and Co in the Dy_4CoCd structure type (ICSD 417044). We were able to locate hydrogen atoms in this cell at eleven different Wyckoff positions: 4*b* (0.5, 0; 0), 16*e* (0.7018; 0.2018; 0.2018), 24*g* (0.25; 0.0952; 0.25), 24*f* (0.0765; 0; 0), 48*h* (0.6255; 0.1255; 0.2969), 48*h* (0.6054; 0.1054; 0.2232), 48*h* (0.6425; 0.1425; 0.0404), 48*h* (0.86997; 0.1303; 0.0395), 48*h* (0.5374; 0.0374; 0.3702), 96*i* (0.6312; 0.0391; 0.7562), and 96*i* (0.7984; 0.0093; 0.0837) (TableS11). The shortest

metal-metal distance is $d_{Y2\text{-}Y4}$ = 2.799 Å, which agrees well with discussed above Y_4H_{23} hydride. The Y – H distances in the first coordination sphere of Y are varied from 1.833 Å to 2.079 Å. This compound has hydrogen triatomic units H7-H10-H7 with an intramolecular bond length of 0.9317 Å, which is shorter than the H-H distance in dimers in Y_4H_{23}.

Discussion 2. The procedure of finding possible positions of the hydrogen atoms in the structures of yttrium hydrides

The starting point for search of hydrogen positions in structures of the novel hydrides was the assumption that the symmetry of the hydride phase would be the same as the symmetry of the yttrium lattice. The hydrogen content of each phase was estimated using a linear relationship between the volume per metal atom in hydrides and their stoichiometry (*i.e*. "Retger's law" approximation, Fig. S8). The search of possible hydrogen atoms position(s) was realized with help of the Endeavour™ software. The composition of the compound is considered as fixed, and positions of yttrium atoms are fixed based on the experimental data. In the Endeavour™ software, we employ a two-body Lennard-Jones potential in the form of $V_{LJ}(r) = 4\varepsilon \cdot \left[\left(\frac{\sigma}{r}\right)^{12} - \left(\frac{\sigma}{r}\right)^{6}\right]$, where r is the distance between two interacting particles, ε is the depth of the potential well, and σ is the distance at which the particle-particle potential energy V is zero. The program searches for the position of the hydrogen atoms by minimizing "cost function" that is the sum of the potential defined above. The output of the Endeavour™ program may contain more than one model for the location of hydrogen atoms. Once the structural search in Endeavour™ is completed, the predicted models are validated using DFT calculations, which involve structural relaxation, verifying their dynamical stability, and the position of the phases on the convex hull in the Y-H binary system. This approach was validated by testing known *cF*4-YH_3 yttrium hydride, and the obtained results were consistent with the literature

The procedure is illustrated below using the novel hydride *cF*80-Y_4H_{25} as an example. The first step of the preparation of the structure solution/search involved defining the cell parameter (*a* = 12.4201 Å) and the space group ($F\bar{4}3m$) (Fig. S2a), followed by specifying the composition and positions of the yttrium atoms (Fig. S2b-c, Table S11). Then, we state that no "diffraction data" would be used, and the search would be conducted solely through "energy" minimization (Fig. S2d). In the subsequent step, the appropriate potential was selected (Fig. S2e). As a result, the program generated a list of the best structural candidates, all of which were validated through DFT calculations. The optimal structure of the phase should meet the following criteria: the difference

in the calculated by DFT unit cell parameters, atomic coordinates, and the calculated pressure should be minimal compared to the experimental values (the numerical criteria for agreement of different characteristics of calculated and experimental data are not strict, and choice of the best or acceptable models in this work is subjective). Fig. S2g illustrates the DFT-relaxed structure for the chosen model from Endeavour, which exhibited a good agreement between theory and experiment (Table S11): for a fixed unit cell volume, the difference between the experimental (171 GPa) and the calculated (168.6 GPa) pressures is ~1.4%; the smallest and the average Y-Y distance in the initial (2.881 Å and 3.208 Å) and in DFT-relaxed (2.811 Å and 3.241 Å) models are very close.

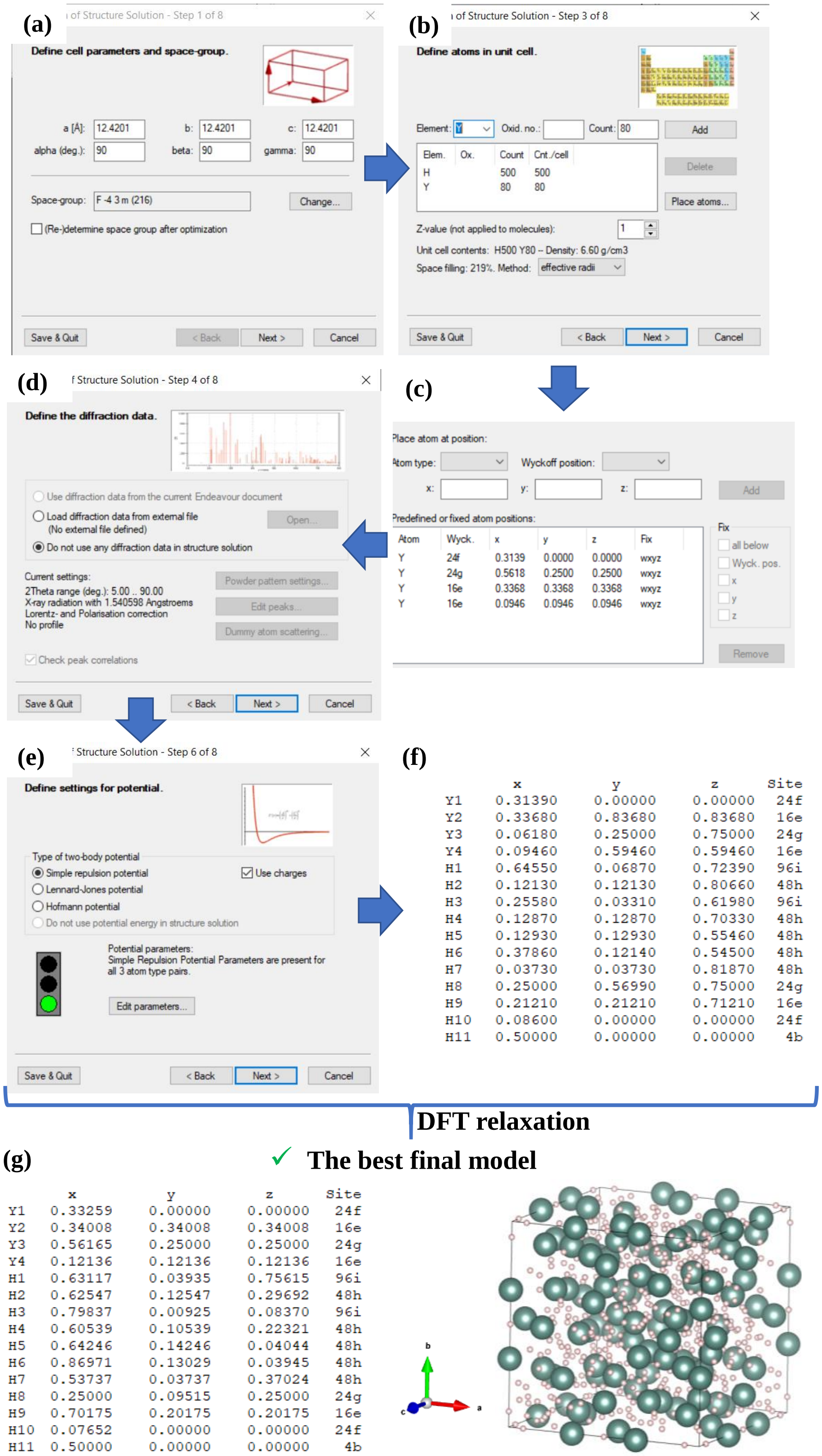

(f)

	x	y	z	Site
Y1	0.31390	0.00000	0.00000	24f
Y2	0.33680	0.83680	0.83680	16e
Y3	0.06180	0.25000	0.75000	24g
Y4	0.09460	0.59460	0.59460	16e
H1	0.64550	0.06870	0.72390	96i
H2	0.12130	0.12130	0.80660	48h
H3	0.25580	0.03310	0.61980	96i
H4	0.12870	0.12870	0.70330	48h
H5	0.12930	0.12930	0.55460	48h
H6	0.37860	0.12140	0.54500	48h
H7	0.03730	0.03730	0.81870	48h
H8	0.25000	0.56990	0.75000	24g
H9	0.21210	0.21210	0.71210	16e
H10	0.08600	0.00000	0.00000	24f
H11	0.50000	0.00000	0.00000	4b

(g)

	x	y	z	Site
Y1	0.33259	0.00000	0.00000	24f
Y2	0.34008	0.34008	0.34008	16e
Y3	0.56165	0.25000	0.25000	24g
Y4	0.12136	0.12136	0.12136	16e
H1	0.63117	0.03935	0.75615	96i
H2	0.62547	0.12547	0.29692	48h
H3	0.79837	0.00925	0.08370	96i
H4	0.60539	0.10539	0.22321	48h
H5	0.64246	0.14246	0.04044	48h
H6	0.86971	0.13029	0.03945	48h
H7	0.53737	0.03737	0.37024	48h
H8	0.25000	0.09515	0.25000	24g
H9	0.70175	0.20175	0.20175	16e
H10	0.07652	0.00000	0.00000	24f
H11	0.50000	0.00000	0.00000	4b

Fig. S2. Schematic step-by-step illustration of the procedure of finding the models of the arrangement of the hydrogen atoms in the structures of novel hydrides

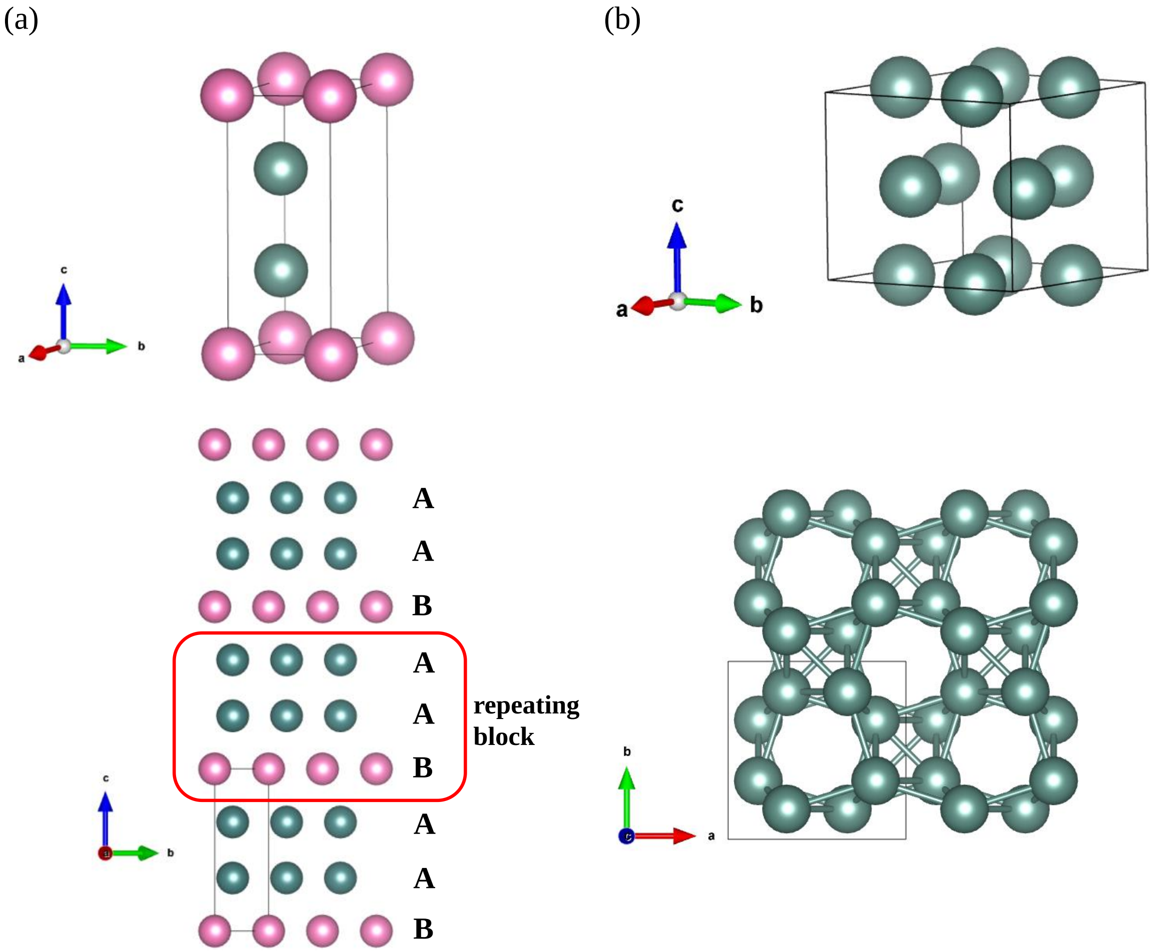

Fig. S3. Crystal structure of the hexagonal *hP*3-Y-II and tetragonal *tI*8-Y yttrium allotropes. (a) Top: the unit cell of *hP*3 Y-II; Y1 and Y2 atoms are marked as pink and green balls, respectively. Bottom: A view of the structure of *hP*3-Y-II along the *a* direction, showing the layer stacking in a '..AAB..' block-sequence pattern along the *c* direction. (b) Top: a unit cell of *tI*8-Y. Yttrium atoms are marked as green balls. Bottom: A view of the structure of *tI*8-Y along the *c* direction, highlighting the channels.

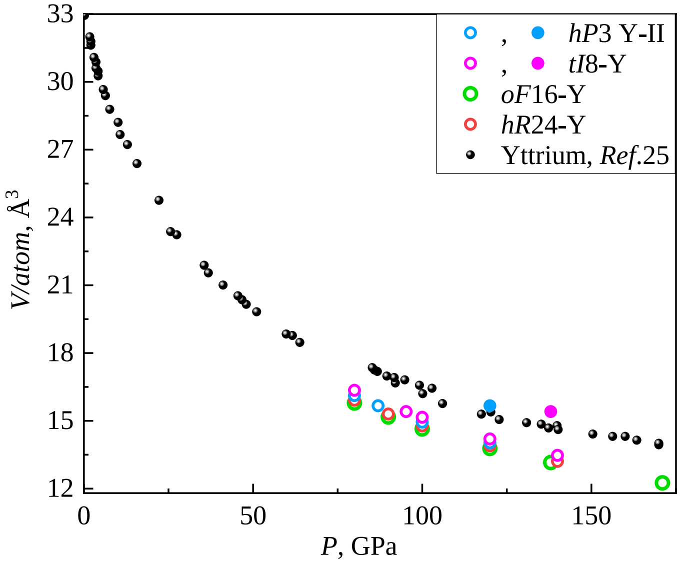

Fig. S4. The experimental and DFT-calculated pressure dependence of the volume per Y atom (*V/atom)* for Y allotropes in a pressure range of 1 bar to 170 GPa. The data for the new hexagonal *hP*3-Y-II and tetragonal *tI*8-Y allotropes as well as for the known *oF*16-Y and *hR*24-Y phases, are shown in blue, pink, green, and red circles, respectively. The pressure dependence of the volume per Y atom for yttrium allotropes, published in *Ref. (34)*, is shown in black circles. The experimental values are shown as solid symbols, while open symbols represent the DFT-calculated values.

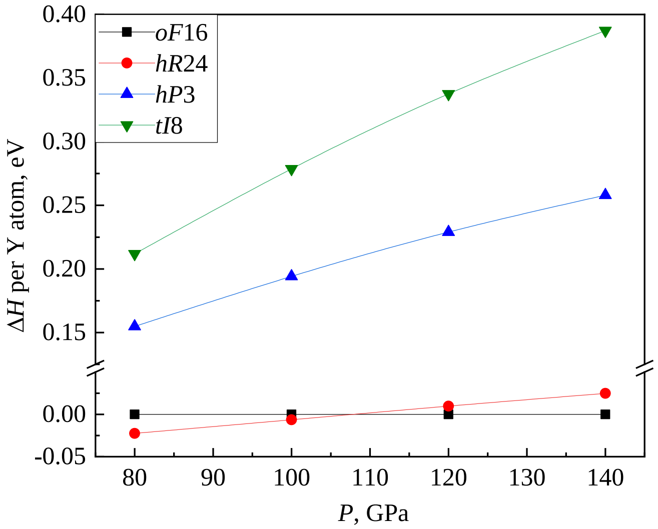

Fig. S5. Results of DFT calculated enthalpy difference between a previously known yttrium allotrope *hR*24-Y, synthesized in this work novel *hP*3-Y-II and *tI*8-Y yttrium allotropes, and previously known *oF*16-Y as a function of pressure: $\Delta H = H_{\mathrm{i}} - H_{oF16\text{-Y}}$.

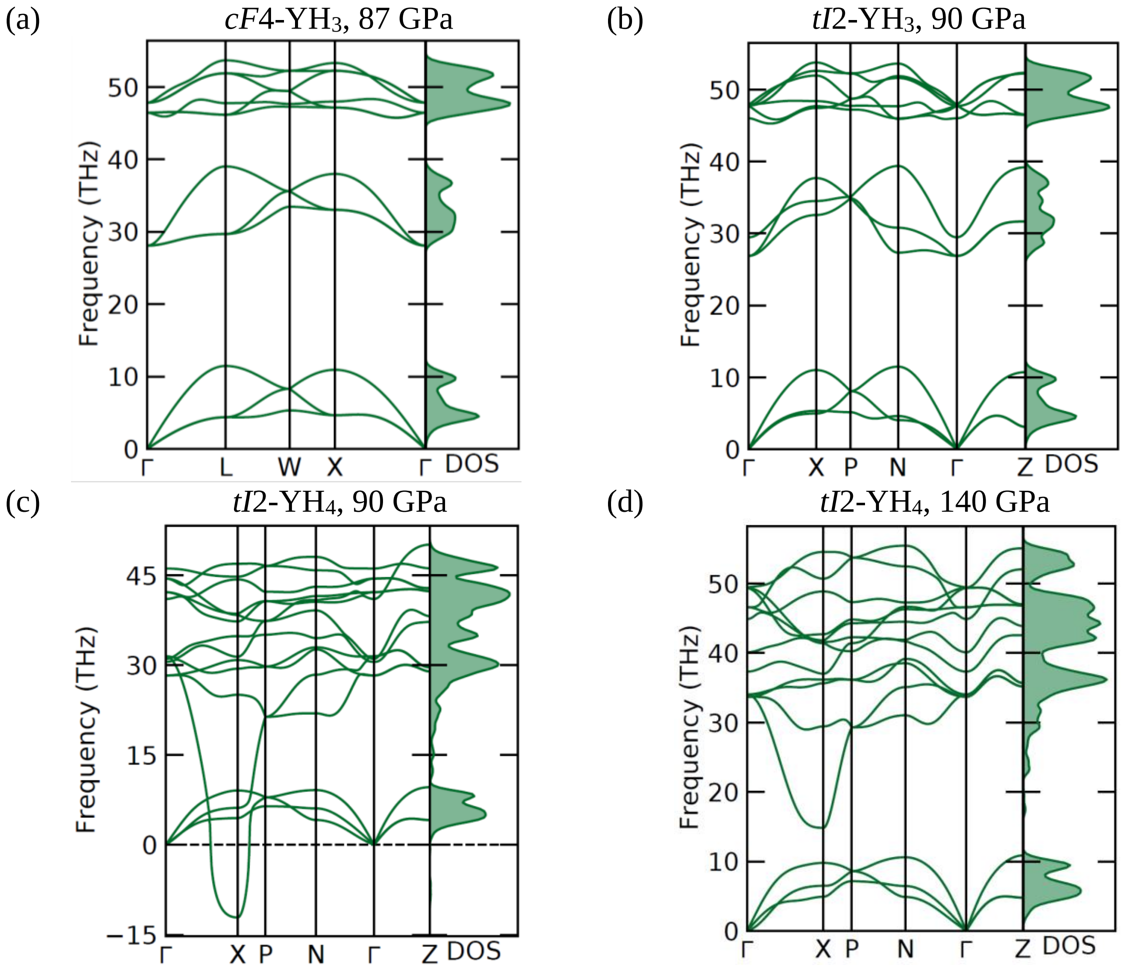

Fig. S6. Phonon dispersion curves calculated along high-symmetry directions in the Brillouin zone, and resulting phonon density of states: (a) for *cF*4-YH_3 at 87 GPa; (b) for *tI*2-YH_3 at 90 GPa (b); (c) for *tI*2-YH_4 at 90GPa and (d) for *tI*2-YH_4 at 140 GPa.

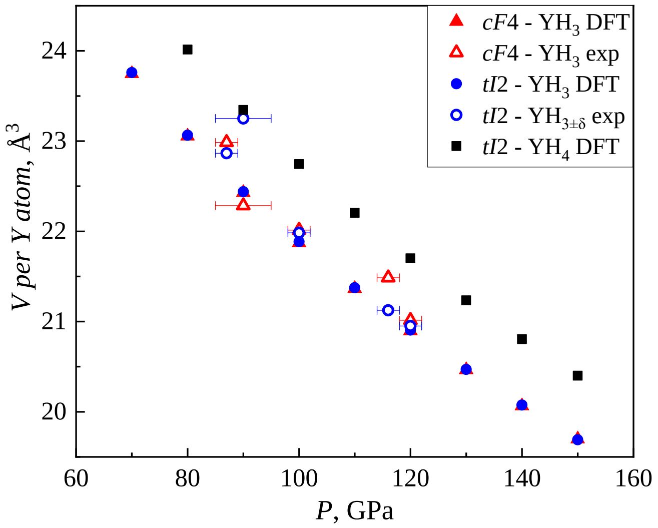

Fig. S7. The pressure dependence of the volume per yttrium atom for yttrium hydrides in a range of 70-150 GPa. The data for *cF*4-YH_3 and *tI*2-$YH_{3\pm\delta}$, are shown in red triangles and blue circles, respectively; open and solid symbols correspond to the experimental and DFT-calculated values, respectively. Black solid squares represent the DFT-calculated values for *tI*2-YH_4.

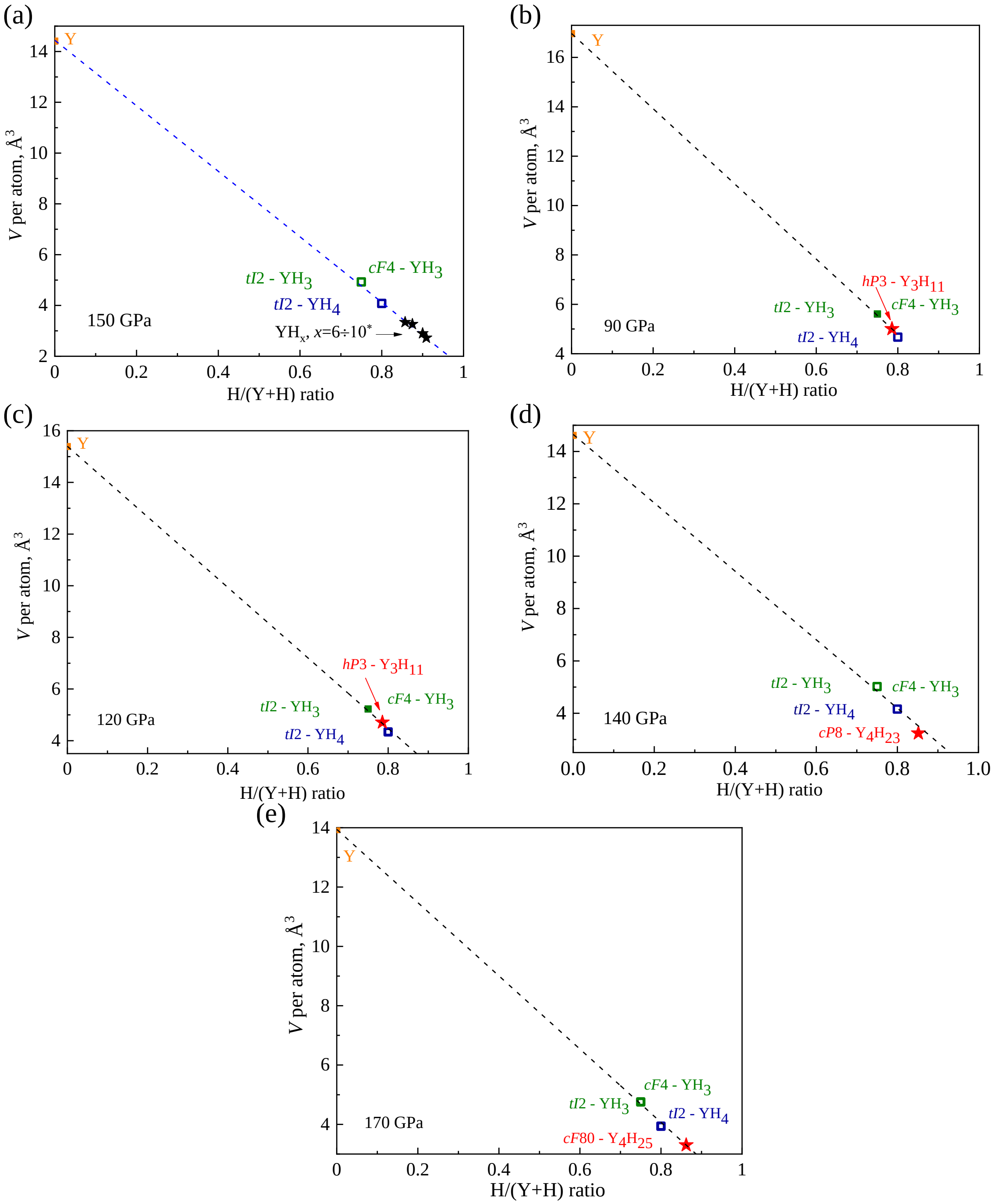

Fig. S8. The illustration of the "Retger's law" approximation for Y-H compounds. The relation between the volume per atom and the hydrogen content is given for (a) 150 GPa, (b) 90 GPa, (c) 120 GPa, (d) 140 GPa, and (e) 170 GPa. The open symbols represent the *P*-*V* data resulting from DFT calculations, solid symbols represent experimental values. The dotted line is the linear fit of the data for Y (*34*), YH_3 and YH_4. The data from (*8*, *7*, *24*) on predicted hydrides YH_x (x=6..10) at 150 GPa are marked as black stars; this data perfectly fit the common trend. The linear fit can be described with the following equations (V_{at} = *V* per atom; *r* = n/(n+m) for each Y_mH_n compound):
(a) $V_{at} = 14.44 - 12.90 \cdot r$; (b) $V_{at} = 16.97 - 15.22 \cdot r$; (c) $V_{at} = 15.39 - 13.66 \cdot r$;
(d) $V_{at} = 14.65 - 13.07 \cdot r$; (e) $V_{at} = 13.94 - 12.35 \cdot r$

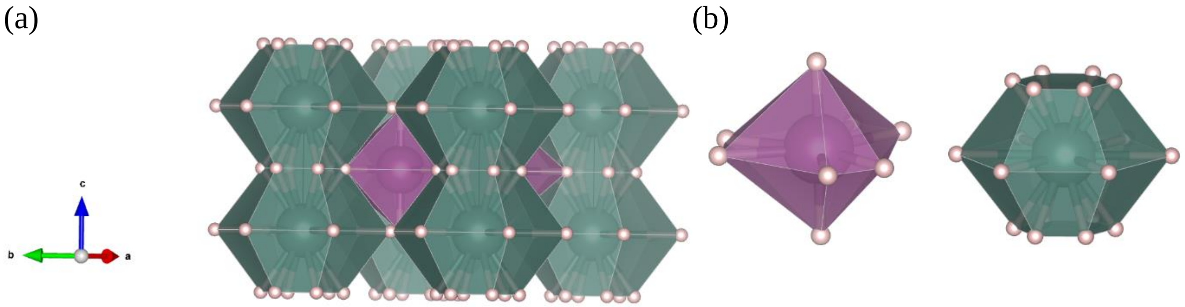

Fig. S9. A polyhedral model of the crystal structure of *hP*3-Y_3H_{11}. (a) A unit cell with the Y atoms shown as violet (Y1) and green (Y2), and hydrogen atoms as light pink balls. (b) Coordination environment of Y1 (violet) and Y2 (green) atoms.

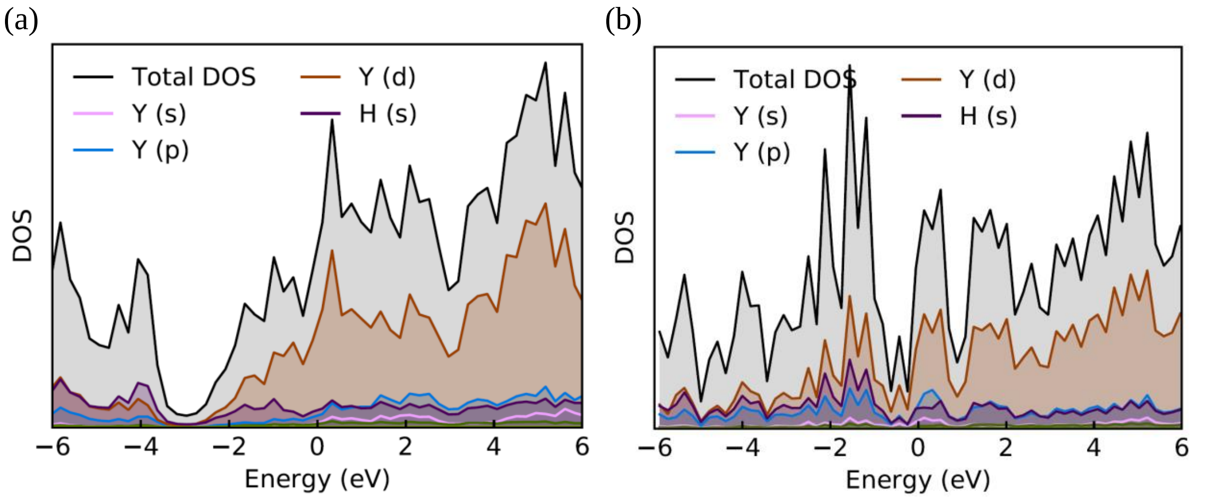

Fig. S10. Electronic density of states for novel high-pressure yttrium hydrides *cP*8-Y_4H_{23} and *cF*80-Y_4H_{25}. (a) The eDOS for *cP*8-Y_4H_{23} at the experimental pressure of 138 GPa. (b) The eDOS for *cF*80-Y_4H_{25} at the experimental pressure of 171 GPa. The Fermi energy level was set to 0 eV.

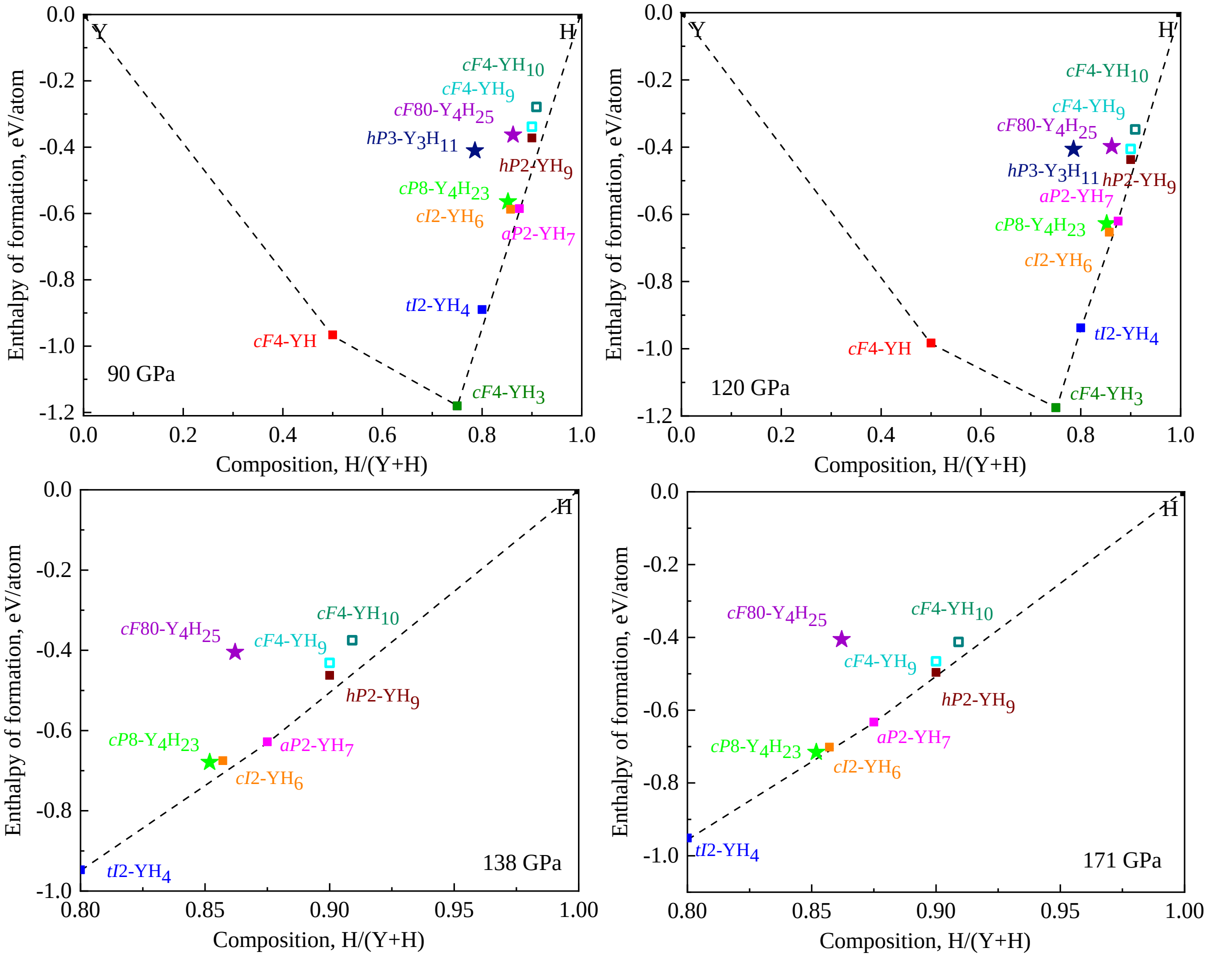

Fig. S11. The calculated convex hull for known binary yttrium hydrides at 90, 120, 138, and 171 GPa. The solid symbols represent phases which were observed experimentally, while open symbols are for predicted phases. The compounds which were synthesized in this work are marked with solid stars. The phases, which lie on the convex hull (black dashed line) are thermodynamically stable.

(a) (b)

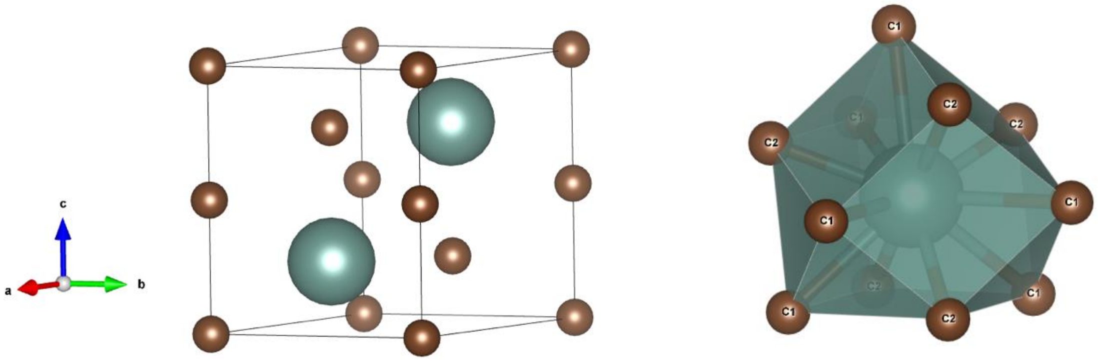

Fig. S12. Crystal structure of yttrium carbide *P*6$_3$/*mmc* YC_2. (a) A unit cell of the structure with the Y atoms shown as green and carbon atoms as brown balls. (b) The Y(C1)$_6$(C2)$_5$ polyhedron.

Table S1: The details of the DAC experiments* and the lists of obtained phases.

	Reagents	*P* (GPa)	Products of the reaction
DAC1	Y in paraffin oil	87(2)	*tI*2-YH_3; *cF*4-YH_3; *hP*3-Y_3H_{11}; YC_2
		116(2)	*tI*2-YH_3; *cF*4-YH_3
DAC2	Y in paraffin oil	100(2)	*tI*2-YH_3; *cF*4-YH_3
DAC3	Y in paraffin oil	120(2)	*tI*2-YH_3; *cF*4-YH_3; YC_2
DAC4	Y in paraffin oil	138(2)	*cP*8-Y_4H_{23}; *hP*26-$Y_{13}H_{75}$; *tI*8-Y
		171(2)	*cF*80-Y_4H_{25}
DAC5	Y in NH_3BH_3	90(5)	*tI*2-YH_3; *cF*4-YH_3; *hP*3-Y_3H_{11}
		120(5)	*hP*3-Y_3H_{11}; *hP*2-Y_2H_9; *hP*3-Y-II

* The maximum temperature during laser-heating was the same within the error for all experiments (T = 3000(200) K)

Table S2: Crystal structure and refinement details of novel HP-HT *hP*3-Y-II yttrium allotrope, obtained in DAC5 at 120 GPa. The corresponding cif file can be accessed from the CCDC database using the identifier CSD 2295636.

Phase			*hP*3-Y-II (experiment)	*hP*3-Y-II (theory)
P, GPa			120(5)	86.9
Space group, *Z*			$P\bar{6}m2$, 3	$P\bar{6}m2$, 3
a, Å			2.773(4)	2.7494
c, Å			7.06(2)	7.1801
V, $Å^3$			47.0(2)	47.0
V, $Å^3$ / Y atom			15.667	15.667
R_{int}, %			3.66	-
Number of measured/independent reflections ($I \geq 3\sigma$)			82 / 50 (49)	-
Number of refined parameters			6	-
R_1, wR_2, % ($I \geq 3\sigma$)			5.7, 13.39	-
$\Delta\rho_{min}$, $\Delta\rho_{max}$, e$Å^{-3}$			1.7, -2.1	-
Wyckoff site and fractional atomic coordinates (x; y; z):	Y1	1*f*	2/3; 1/3; 0.5	2/3; 1/3; 0.5
	Y2	2*h*	1/3; 2/3; 0.1957(8)	1/3; 2/3; 0.17258

Table S3: Crystal structure and refinement details of novel HP-HT *tI*8-Y yttrium allotrope, obtained in DAC4 at 138 GPa. The corresponding cif file can be accessed from the CCDC database using the identifier CSD 2295638.

Phase			*tI*8-Y (experiment)	*tI*8-Y(theory)
P, GPa			138(2)	95.3
Space group			*I*4/*mcm*, 8	*I*4/*mcm*, 8
a, Å			5.3001(10)	5.1116
c, Å			4.391(3)	4.7172
V, $Å^3$			123.35(8)	123.35
V, $Å^3$ / Y atom			15.4188	15.4188
R_{int}, %			8.70	-
Number of measured/independent reflections ($I \geq 3\sigma$)			309 / 106 (79)	-
Number of refined parameters			5	-
R_1, wR_2, % ($I \geq 3\sigma$)			4.55, 9.65	-
$\Delta\rho_{min}$, $\Delta\rho_{max}$, $eÅ^{-3}$			2.1, -2.6	-
Wyckoff site and fractional atomic coordinates (*x*; *y*; *z*):	Y1	8*h*	0.1703(1); 0.6703(1);0.5	0.1817; 0.6817;0.5

Table S4: Crystal structure and refinement details of *cF*4-YH_3 ($Fm\bar{3}m$) for all experimental pressure points at which it was observed.

DAC number	DAC1	DAC2	DAC1	DAC3	DAC5*
P, GPa	87(2)	100(2)	116(2)	120(2)	90(5)
a, Å	4.5133(18)	4.4488(9)	4.4129(17)	4.3872(13)	4.467(3)
V, $Å^3$	91.94(6)	88.05(3)	85.94(6)	84.44(4)	89.14(11)
V, $Å^3$ / Y atom	22.985	22.013	21.485	21.110	22.285
R_{int}, %	4.3	2.09	3.09	3.83	-
Number of measured/independent reflections ($I \geq 3\sigma$)	144 / 35 (35)	57 / 21 (21)	57 / 18 (18)	63 / 22 (22)	-
Number of refined parameters	2	2	2	2	-
R_1, wR_2, % ($I \geq 3\sigma$)	4.41, 10.1	4.76, 10.9	5.8, 14.1	4.43, 10.25	-
$\Delta\rho_{min}$, $\Delta\rho_{max}$, $eÅ^{-3}$	3.0, -1.1	2.5, -1.8	1.5, -2.5	1.1, -1.7	-

* The unit cell parameters and volumes were obtained from powder XRD data.

Table S5: Crystal structure and refinement details of *tI*2-YH_3 (*I*4/*mmm*) at all experimental pressures.

DAC number	DAC1	DAC2*	DAC1	DAC3	DAC5*
P, GPa	87(2)	100(2)	116(2)	120(2)	90(5)
a, Å	3.2013(16)	3.1479(5)	3.0888(11)	3.123(2)	3.2292(11)
c, Å	4.465(4)	4.4371(10)	4.4285(19)	4.301(3)	4.5022(17)
V, Å^3	45.75(6)	43.968(17)	42.25(3)	41.9(3)	46.95(3)
V, Å^3 / Y atom	22.875	21.984	21.125	20.95	23.25
R_{int}, %	3.84	-	1.3	2.4	-
Number of measured/independent reflections (I ≥ 3σ)	142 / 54 (54)	-	105 / 59 (58)	100 / 42 (42)	-
Number of refined parameters	3	-	3	3	-
R_1, wR_2, % ($I \geq 3\sigma$)	3.1, 6.8	-	5.89, 15.04	4.22, 10.5	-
$\Delta\rho_{min}$, $\Delta\rho_{max}$, eÅ^{-3}	-3.1, 2.4	-	3.4, -3.3	2.5, -1.8	-

* The unit cell parameters and volumes were obtained from powder XRD data.

Table S6: The values of the Y-Y distances in novel yttrium hydrides and in yttrium allotropes known in the literature, shown at 90, 120, 138, and 171 GPa.

Phase	Pressure, GPa	Minimal Y-Y distance, Å	Y-Y distance in Y metal, Å
Y_3H_{11}	90	2.9578(2)	2.6334 ($R\bar{3}m$ – Y)
Y_2H_9	120	3.0870(12)	2.5395 (*Fddd* – Y)
$Y_{13}H_{75}$	138	2.8816(4)	2.5099 (*Fddd* – Y)
Y_4H_{23}	138	2.7908(5)	2.5099 (*Fddd* – Y)
Y_4H_{25}	171	2.8806(3)	2.4452 (*Fddd* – Y)

Table S7: Crystal structure and refinement details of novel yttrium hydride *hP*3-Y_3H_{11} (*P*6/*mmm*) at all experimental pressures. The corresponding cif file at 120 GPa can be accessed from the CCDC database using the identifier CSD 2295619.

DAC number	DAC1	DAC5	DAC5
P, GPa	87(2)	90(5)	120(5)
a, Å	5.123(3)	5.140(5)	4.978(3)
c, Å	3.083(3)	3.106(3)	3.0784(15)
V, $Å^3$	70.07(10)	71.06(12)	66.06(9)
V, $Å^3$ / Y atom	23.357	23.687	22.02
R_{int}, %	7.22	4.61	8.79
Number of measured/independent reflections (I ≥ 3σ)	455 / 132 (78)	106 / 42 (21)	147 / 51 (22)
Number of refined parameters	5	5	5
R_1, wR_2, % ($I \geq 3\sigma$)	6.39, 17.1	8.60, 21.5	4.68, 14.45
$\Delta\rho_{min}$, $\Delta\rho_{max}$, $eÅ^{-3}$	3.8, -2.9	2.8, -2.5	1.4, -2.0

Table S8: The DFT-relaxed structure of a novel HP yttrium hydride *hP*3-Y_3H_{11} at 90 and 120 GPa.

Crystal phase			*hP*3-Y_3H_{11} (theory)	*hP*3-Y_3H_{11} (theory)
Space group, *Z*			*P*6/*mmm*, 1	*P*6/*mmm*, 1
a (Å)			4.99089	4.79494
c (Å)			3.30781	3.21793
V ($Å^3$)			71.06	66.06
Pressure *P* (GPa)			97.2	123.2
Wyckoff site and fractional atomic coordinates (*x*; *y*; *z*):	Y1	2*d*	2/3; 1/3; 0.5	2/3; 1/3; 0.5
	Y2	1*a*	0; 0; 0	0; 0; 0
	H1	6*k*	0.24050; 0; 0.5	0.23539; 0; 0.5
	H2	3*f*	2/3; 1/3; 0	0.5; 0; 0
	H3	2*c*	0.5; 0; 0	0; 0; 0.5

Table S9: Crystal structure and refinement details for the novel yttrium hydrides *cP*8-Y_4H_{23}, *hP*26-$Y_{13}H_{75}$, *cF*80-Y_4H_{25}, and *hP*2-Y_2H_9 at different experimental pressures. The corresponding cif file can be accessed from the CCDC database using the identifiers CSD 2295644, 2295626, 2295645, and 2295620, respectively.

Phase	*cP*8-Y_4H_{23}	*hP*26-$Y_{13}H_{75}$	*cF*80-Y_4H_{25}	*hP*2-Y_2H_9
DAC number	DAC4	DAC4	DAC4	DAC5
P, GPa	138(2)	138(2)	171(2)	120(5)
Space group	$Pm\bar{3}n$	$P6_3/mmc$	$F\bar{4}3m$	$P6_3/mmc$
a, Å	5.5815(8)	8.9730(13)	12.4201(14)	3.162(3)
c, Å	-	8.9085(8)	-	4.958(2)
V, $Å^3$	173.88(7)	621.17(19)	1915.9(4)	42.94(9)
V, $Å^3$ / Y atom	21.735	23.891	23.949	21.47
R_{int}, %	9.83	9.67	9.98	9.16
Number of measured/independent reflections ($I \geq 3\sigma$)	555 / 90 (67)	3505 / 974 (601)	1139 / 371 (210)	70 / 28 (20)
Number of refined parameters	4	17	15	3
R_1, wR_2, % ($I \geq 3\sigma$)	4.84, 10.81	4.34, 8.11	5.71, 12.43	8.24, 16.98
$\Delta\rho_{min}$, $\Delta\rho_{max}$, $eÅ^{-3}$	4.1, -2.5	3.0, -2.9	1.8, -1.9	2.0, -1.6

Table S10: Crystallographic data for a novel HP yttrium hydride *cP*8-Y_4H_{23} at 138 GPa in comparison to the corresponding DFT-relaxed structure.

Crystal phase			Y_4H_{23} (experiment)	Y_4H_{23} (theory)
Space group, *Z*			$Pm\bar{3}n$, 2	$Pm\bar{3}n$, 2
a (Å)			5.5815(8)	5.5815
V ($Å^3$)			173.88(7)	173.88
Pressure *P* (GPa)			138 (2)	147.2
Wyckoff site and fractional atomic coordinates (*x*; *y*; *z*):	Y1	6*d*	0.25; 0.5; 0	0.25; 0.5; 0
	Y2	2*a*	0; 0; 0	0; 0; 0
	H1*	6*c*	-	0.25; 0; 0.5
	H2*	16*i*	-	0.2010; 0.2010; 0.2010
	H3*	24*k*	-	0; 0.3103; 0.1492

* The hydrogen atoms were placed according to the structural data for isostructural hydrides M_4H_{23} (*M* = Ba, La, and Eu).

Table S11: Experimentally determined crystallographic data for the novel HP yttrium hydride *cF*80-Y_4H_{25} at 171 GPa in comparison to the corresponding DFT-relaxed structure.

Crystal phase			Y_4H_{25} (experiment)	Y_4H_{25} (theory)
Space group, *Z*			$F\bar{4}3m$, Z = 20	$F\bar{4}3m$, Z = 20
a (Å)			12.4201(11)	12.4201
V (Å^3)			1915.9(4)	1915.9
Pressure *P* (GPa)			171 (2)	168.6
Wyckoff site and fractional atomic coordinates (*x*; *y*; *z*):	Y1	24*f*	0.3130; 0; 0	0.3326; 0; 0
	Y2	16*e*	0.3368; 0.3368; 0.3368	0.3401; 0.3401; 0.3401
	Y3	24*g*	0.5616; 0.25; 0.25	0.5617; 0.25; 0.25
	Y4	16*e*	0.0943; 0.0943; 0.0943	0.1214; 0.1214; 0.1214
	H1	96*i*	-	0.6312; 0.0391; 0.7562
	H2	48*h*	-	0.6255; 0.1255; 0.2969
	H3	96*i*	-	0.7984; 0.0093; 0.0837
	H4	48*h*	-	0.6054; 0.1054; 0.2232
	H5	48*h*	-	0.6425; 0.1425; 0.0404
	H6	48*h*	-	0.8697; 0.1303; 0.0395
	H7	48*h*	-	0.5374; 0.0374; 0.3702
	H8	24*g*	-	0.25; 0.0952; 0.25
	H9	16*e*	-	0.7018; 0.2018; 0.2018
	H10	24*f*	-	0.0765; 0; 0
	H11	4*b*	-	0.5; 0; 0

Table S12: Experimentally determined crystallographic data for a novel HP yttrium carbide YC_2 at 120 GPa in comparison to the corresponding DFT-relaxed structure. The corresponding cif file can be accessed from the CCDC database using the identifier CSD 2295639.

Crystal phase			YC_2 (experiment)	YC_2 (theory)
Space group, *Z*			$P6_3/mmc$, 2	$P6_3/mmc$, 2
a (Å)			3.6000(17)	3.6000
c (Å)			4.439(8)	4.439
V (Å^3)			49.82(13)	49.82
Pressure *P* (GPa)			120 (2)	127.3
Wyckoff site and fractional atomic coordinates (*x*; *y*; *z*):	Y1	2*c*	1/3; 2/3; 1/4	1/3; 2/3; 1/4
	C1	2*a*	0; 0; 0	0; 0; 0
	C2	2*d*	1/3; 2/3; 3/4	1/3; 2/3; 3/4
Number of measured/independent reflections ($I \geq 3\sigma$)			129/ 36 (32)	-
R_{int}			0.0675	-
Final *R* indexes (all)			R_1 = 0.0506; wR_2 = 0.915	
Final *R* indexes ($I \geq 3\sigma$)			R_1 = 0.0396; wR_2 = 0.0869	-
$\Delta\rho_{min}$, $\Delta\rho_{max}$, eÅ^{-3}			-0.7, 0.5	
Number of refined parameters			4	-

Data S1. Crystallographic information file for *hP*3-Y-II.
Data from single-crystal X-ray diffraction experiments at 120 GPa for *hP*3-Y.

Data S2. Crystallographic information file for *tI*8-Y.
Data from single-crystal X-ray diffraction experiments at 138 GPa for *tI*8-Y.

Data S3. Crystallographic information file for Y_2H_9.
Data from single-crystal X-ray diffraction experiments at 120 GPa for Y_2H_9.

Data S4. Crystallographic information file for Y_3H_{11}.
Data from single-crystal X-ray diffraction experiments at 120 GPa for Y_3H_{11}.

Data S5. Crystallographic information file for Y_4H_{23}.
Data from single-crystal X-ray diffraction experiments at 138 GPa for Y_4H_{23}.

Data S6. Crystallographic information file for Y_4H_{25}.
Data from single-crystal X-ray diffraction experiments at 171 GPa for Y_4H_{25}.

Data S7. Crystallographic information file for $Y_{13}H_{75}$.
Data from single-crystal X-ray diffraction experiments at 138 GPa for $Y_{13}H_{75}$.

Data S8. Crystallographic information file for YC_2.
Data from single-crystal X-ray diffraction experiments at 120 GPa for YC_2.
